# Quantitative determination of *sn*-positional phospholipid isomers in MS^n^ using silver cationization

**DOI:** 10.1007/s00216-022-04173-6

**Published:** 2022-06-22

**Authors:** Johan Lillja, Ingela Lanekoff

**Affiliations:** Department of Chemistry – BMC (576), Uppsala University, 751 23 Uppsala, Sweden

**Keywords:** Sn-isomer, Phospholipid isomer, Tandem mass spectrometry, MS^n^, Quantitative, Nano-DESI, Mass spectrometry imaging, Silver, Silver cationization

## Abstract

Glycerophospholipids are one of the fundamental building blocks for life. The acyl chain connectivity to the glycerol backbone constitutes different *sn*-positional isomers, which have great diversity and importance for biological function. However, to fully realize their impact on function, analytical techniques that can identify and quantify *sn*-positional isomers in chemically complex biological samples are needed. Here, we utilize silver ion cationization in combination with tandem mass spectrometry (MS^n^) to identify *sn*-positional isomers of phosphatidylcholine (PC) species. In particular, a labile carbocation is generated through a neutral loss (NL) of AgH, the dissociation of which provides diagnostic product ions that correspond to acyl chains at the *sn*-1 or *sn*-2 position. The method is comparable to currently available methods, has a sensitivity in the nM–µM range, and is compatible with quantitative imaging using mass spectrometry in MS^4^. The results reveal a large difference in isomer concentrations and the ion images show that the *sn*-positional isomers PC 18:1_18:0 are homogeneously distributed, whereas PC 18:1_16:0 and PC 20:1_16:0 show distinct localizations to sub-hippocampal structures.

**Graphical abstract:**

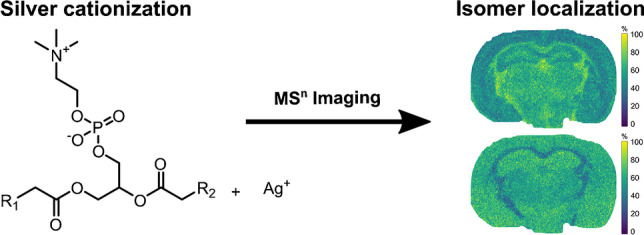

**Supplementary Information:**

The online version contains supplementary material available at 10.1007/s00216-022-04173-6.

## Introduction

Glycerophospholipids consist of a glycerol backbone to which fatty acyl side chains and a phosphate head group are bound. Glycerophospholipids have several functions in cells including being the major components of cellular membranes, essential in the induction of cell signaling, and important as precursors and storage centers for smaller signaling molecules [1, 2]. The phosphate head group is located at the *sn*-3 position of the glycerol backbone and the two acyl chains in the *sn*-1 and *sn*-2 positions. The composition of the head group affects the curvature of the cell membrane, specifies its location to the outer and inner leaflets of the cell membrane, and determines protein-specific interactions and signaling pathways [3–5]. The acyl chains can be branched, and vary in length and degree of unsaturation, which alters membrane fluidity, enables cellular adaptation to temperature changes, interacts with membrane proteins, and is essential in maintaining cellular homeostasis [6–9]. Furthermore, the acyl chain connectivity to the *sn*-1 or the *sn*-2 position has been shown to alter the rate of cholesterol depletion by β-cyclodextrins in liposomes [10]. Thus, the ability to determine isomer specificity is important to understand their effect on membrane properties and biological function.

Mass spectrometry of glycerophospholipids enables identification based on their mass-to-charge value (*m/z*), and further structural elucidation can be provided by the use of tandem mass spectrometry, MS^2^, and MS^n^ [11]. In particular, the head group of glycerophospholipids is readily determined in MS^2^ by identifying and/or scanning for the specific head group product ions or neutral losses (NL) [11, 12]. The acyl chain composition and position on the glycerol backbone are more challenging to determine by tandem mass spectrometry. The acyl chain composition can be anticipated based on neutral losses or carboxylate anions in the negative ion mode, but to determine the connectivity to the glycerol backbone fragmentation efficiency for the product ions usually needs to be rationalized [12]. Specifically, it has been shown that the carboxylate anion of an acyl chain is preferential at the *sn*-2 position for PC lipids [13, 14]. Moreover, Ekroos et al. [14] showed that in some cases the *sn* position of acyl chains in PC species could be determined as acetate or chloride adducts in the negative ion mode by generating demethylated lysoPC product ions in MS^3^. Other experimental approaches to deduce the connectivity of the acyl chains with mass spectrometry include ozone-induced dissociation (OzID) [15], ion/ion reactions [16], electron-based fragmentation [17], radical directed fragmentations [18], Paternò-Büchi derivatizations online and offline [19], ultraviolet photodissociation (UVPD) [20, 21], and ion mobility spectrometry (IMS) [22]. Interestingly, by using OzID, the specific lipid isomers PC 16:0/20:1 and PC 16:0/18:1 were shown to localize in the white matter of the mouse brain by MSI [23]. It has also been found that the acyl chain connectivity and double bond position in the acyl chain are expressed differently in breast cancer cell lines, lung cancer tissue, and type 2 diabetes using Paternö-Büchi reactions [19]. The acyl chain distributions are often attributed to differential activity and expression of phospholipase and acyltransferase enzymes, and detection of *sn*-positional isomers could therefore reveal details of lipid metabolism [24]. However, existing analytical approaches generally require specialized equipment, which can limit the adaptation of the techniques for common users.

To reduce the need for specialized equipment, silver ions have been used to improve both the sensitivity and selectivity in lipid analysis [25–27]. Silver ions interact with the π-moieties of alkene structures, which is utilized in silver chromatography to separate lipid species based on their degree of unsaturation [28–30]. The interaction between Ag^+^ and π-moieties has also been utilized as an ionization reagent for mass spectrometric experiments, and to obtain information on the molecular structure [25, 27, 29, 31, 32]. Silver ions have been shown to increase the sensitivity of poorly ionizable species, [25, 26] and to induce structural changes in the gas phase to allow for separation by ion mobility spectrometry (IMS) and tandem mass spectrometry [25, 26, 29, 31, 32]. The use of tandem mass spectrometry to discriminate lipid isomers is highly attractive due to its congruency with most modern MS systems capable of collision-induced dissociation (CID). However, current literature on silver ionized CID-activated glycerophospholipids has not previously shown the specificity or applicability in complex mixtures or the application to saturated lipids [29, 31].

In this study, we describe the use of silver for the cationization of saturated and unsaturated acyl chains of glycerophospholipids to pinpoint their positions on the glycerol backbone. We describe a CID fragmentation pathway down to MS^4^ to generate diagnostic product ions for *sn*-positional isomers in complex chemical mixtures through the reduction of the silver ion. Finally, we show that the method is quantitative, and readily adaptable to imaging through silver-doped nanospray desorption electrospray ionization (nano-DESI) MSI to quantitatively map *sn*-positional isomers in the mouse brain.

## Experimental

### Standard solutions and preparation

Monoisotopic silver nitrate was used to reduce spectral complexity and overlaps from species which differ in one double bond [33]. Briefly, a solid sample of ^107^Ag (Trace Sciences International, Richmond Hill, ON, Canada) was dissolved in sub-boiled nitric acid. Excess acid was evaporated and the ^107^AgNO_3_ product was weighted. The ^107^AgNO_3_ was dissolved in 20 mL water from an in-house water purification system (Merck KGaA, Darmstadt, Germany) to a concentration of 1100 ppm, the vial was covered with aluminum foil and stored at 4 °C before use.

Standards and solvents were of analytical grade and purchased from Merck (Darmstadt, Germany); all lipid standards were stored at − 20 °C before analysis. Working standards of PC 18:1/16:0 and PC 16:0/18:1 were prepared gravimetrically and dissolved in 2:1 (v/v) chloroform:methanol. Calibration curves of the two PC isomers were prepared between 0 and 0.5 µM volumetrically and spiked with 1 µM of the opposite isomer in 2:1 (v/v) CHCl_3_:MeOH with 10 ppm ^107^Ag^+^.

### Mass spectrometry

An Orbitrap Velos Pro (Thermo Fisher Scientific, Bremen, Germany) was used in both Fourier transform (FTMS) and ion trap (IT) mode. For the FTMS experiments, a resolution of 100,000 (*m*/Δ*m* at *m/z* 400) was used, and for the IT experiments, the normal scan rate (33,333 Da/s) with 3 microscans was utilized. Tandem mass spectrometry (MS^n^) experiments were performed with an isolation window of 1 Da, normalized collision energy (NCE) 35–40 for all transitions, activation q was set to 0.5, and a 10 ms activation time.

All standards were measured with a direct infusion at a flow rate of 5 μLmin^−1^ using the built-in syringe pump at the mass spectrometer. A 34-gauge stainless steel capillary (Thermo Fisher Scientific, Bremen Germany) was used as the ESI capillary, sheath gas was set to 3 (arb. Unit), auxiliary gas set to 5 (arb. Unit), a potential of 3.5 kV was applied for ESI, the s-lens RF was kept at 65%, and the inlet capillary temperature was kept at 300 °C for all experiments.

A select series cyclic IMS (Waters Corp., Wilmslow, UK) was used for IMS^n^ experiments. The TOF was run in V-mode for all experiments. The wave height of the traveling wave ion mobility cell was set to 25 V for all experiments and 1–10 cycles were tested for the IMS^n^ transitions. The sample was ionized with ESI with the standard ion source which was operated at a flow rate of 0.5 µL min^−1^.

### Mass spectrometry imaging

Mouse brains were acquired from Creative Biolabs (NY, USA) and sectioned to a thickness of 10 µm using a cryo-microtome (Leica Microsystems, Wetzlar, Germany). The sections were thaw-mounted on glass slides and stored at − 80 °C before analysis. All handling of biological tissue was done in accordance with local ethics and safety regulations. Nanospray desorption electrospray ionization (nano-DESI) mass spectrometry imaging (MSI) was set up as described in Lanekoff et al. [34] using fused silica capillary (Polymicro Technologies, USA) with dimensions 150/50 µm (o.d./i.d.) for the primary and secondary capillaries. The extraction solvent was prepared by dissolving the internal standard PC 25:0 to the final concentration of 0.5 µM and 10 ppm ^107^Ag^+^ in 9:1 acetonitrile:methanol. The XYZ stage (Newport, CA, USA) was controlled with a Labview program and moved at 20 µm s^−1^ across the sample in 96 lines with the spacing between the lines set to 75 µm, resulting in two times oversampling and a total runtime of about 16 h [35]. The mass spectrometer was set to acquire a full MS scan in the range of 200–2000 m*/z* using protonated PC 34:1 as lock mass followed by four MS^n^ scan events in the IT with 3 microscans. The scan parameters of the stage combined with the duty cycle for the MS results in an approximate pixel size of 60 × 75 µm. All raw data files were converted to.mzXML using Proteowizard MSConvert [36]. Data processing was done in MATLAB version 2018b (MathWorks, Natick, MA, USA) using in-house scripts.

## Results and discussion

### Diagnostic product ions for sn-positional isomer annotation

**Fig. 1:**
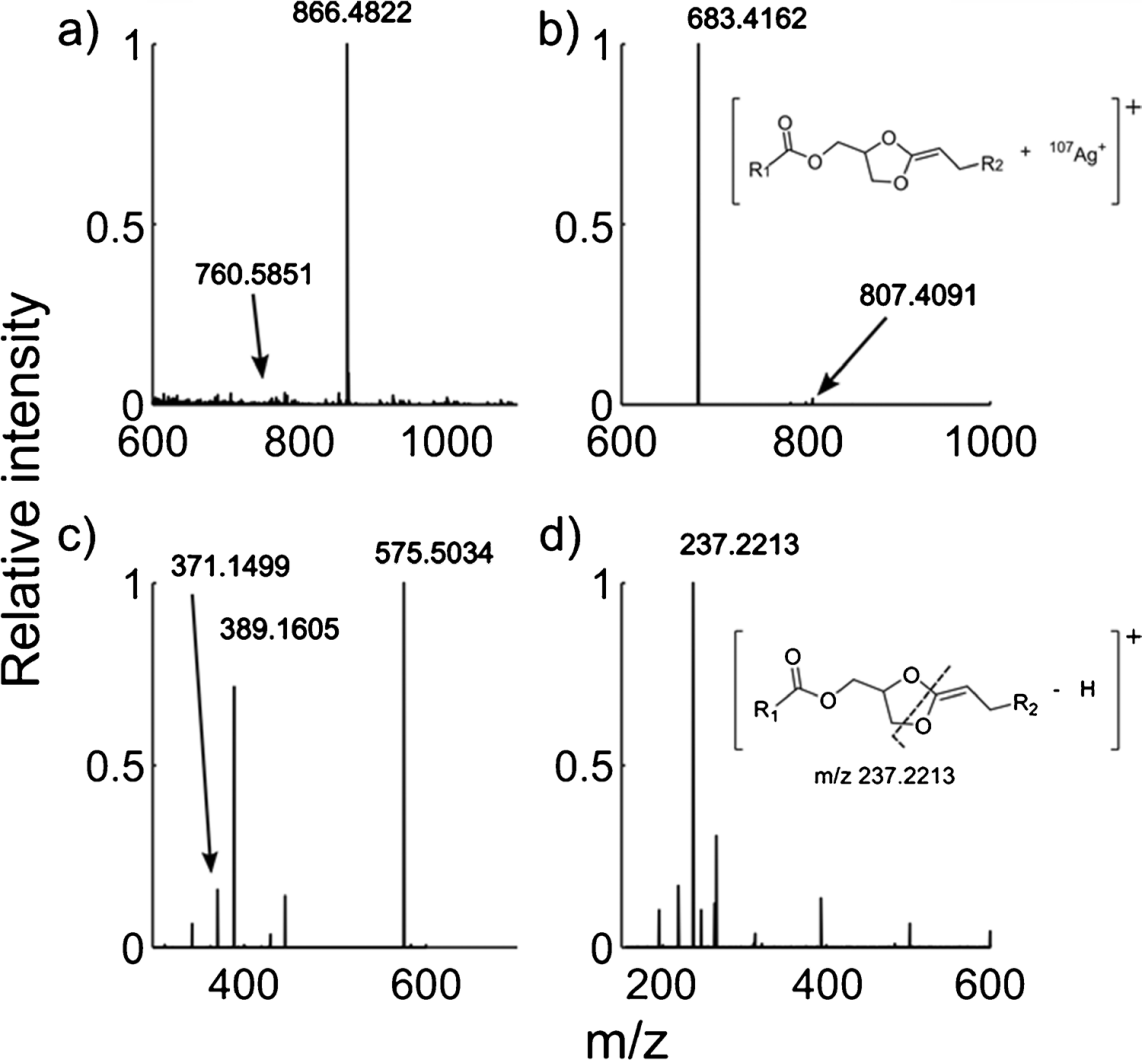
Tandem mass spectrometric transitions of PC 18:1/16:0 at a concentration of 0.5 µM with 10 ppm ^107^Ag^+^ in the solvent. **a** MS^1^ spectrum showing the [M + H]^+^ ion at *m/z* 760.5839 and [M + ^107^Ag]^+^ at *m/z* 866.4822. **b** MS^2^ spectrum of the [M + Ag]^+^ peak giving product ions corresponding to choline loss at *m/z* 807.4091 and head group loss at *m/z* 683.4162. **c** MS^3^ spectrum using *m/z* 683.4162 as precursor ion, where the main product ion at *m/z* 575.5034 corresponds to a NL of AgH and the other annotated ions represent acyl chain losses. **d** MS^4^ of the *m/z* 575.5034 ion giving diagnostic product ions corresponding to the acyl chains 18:1 and 16:0 in the *sn*-1 and *sn*-2 position, respectively

Electrospray ionization tandem mass spectrometry experiments were performed using monoisotopic silver-containing standard solutions of the *sn*-positional isomer pair PC 16:0/18:1 and PC 18:1/16:0. The resulting MS^1^ spectrum in Fig. 1a shows that silver cationization (*m/z* 866.4822) exceeds all other adduct formations, suggesting that silver adduct formation of PC 34:1 is preferred under these experimental conditions. Fragmentation of the [M + ^107^Ag]^+^ precursor ions of PC 16:0/18:1 and PC 18:1/16:0 in MS^2^ produces product ions at *m/z* 807.4091 and *m/z* 683.4162, which correspond to losses of the choline moiety (NL of C_3_H_9_N) and the full head group (NL of C_5_H_14_NPO_4_), respectively (Figs. 1b and S1) [37]. These losses are also observed from other alkali metal adduct ions, including sodium and potassium adducts [37]. The structure of the MS^2^ product ion from alkali metal adducts of glycerolipids consists of a five-membered 1,3-dioxolane ring over the glycerol backbone and a C = C to the adjacent *sn*-2 acyl chain, which has the potential to form unique product ions in MS^3^ (Fig. 1b) [20, 38–40]. This is reported as the necessary precursor ion to determine *sn*-positional isomer structures using UVPD, OzID, and PB methods [19, 20, 41]; however, the product ions generated using silver cationization are vastly different.

Fragmentation of the MS^2^ product ion of silver cationized PC 18:1/16:0 at *m/z* 683.4162 in MS^3^ primarily generates ions corresponding to the fatty acyl (FA) chain. In particular, [FA + Ag]^+^, [FA + Ag-H_2_O]^+^, and [FA + Ag-H_2_O-CO]^+^ are detected, which have been previously suggested as characteristic ions for *sn* connectivity (Fig. 1c) [29]. However, the [FA + ^107^Ag]^+^ product ions are formed from both the *sn*-1 and the *sn*-2 isomers with CID (Fig. S2). Thus, these ions cannot be considered diagnostic for a particular *sn*-positional isomer structure and cannot be used exclusively for annotation. Interestingly, the MS^2^ precursor ion at *m/z* 683.4162 also dissociates with the product ion at *m/z* 575.5034 in MS^3^ (Fig. 1c). This corresponds to a neutral loss of ^107^AgH [29, 31, 40, 42–44], which is confirmed by mass spectrometric measurements of [FA + ^107^Ag]^+^ and [FA + ^109^Ag]^+^ (Fig. S3). This loss is unique for Ag cationization and therefore the ion at *m/z* 575.5034 could provide additional structural insights.

Ion mobility measurements can provide information on the structure of the ion through drift time measurements. The conformation of PC 16:0/18:1 and PC 18:1/16:0 silver adducts in MS^1^, MS^2^, and MS^3^ were analyzed using direct infusion electrospray cyclic ion mobility spectrometry and tandem mass spectrometry (cIMS^n^). The results show that *sn*-positional isomers are baseline separated in cIMS^1^ using 10 passes (Fig. S4a and S4b), similar as shown by differential mobility [31]. Further selection and analysis of individual isomers show that there is a distinct difference in drift time between the silver-containing product ions after the neutral loss of the PC head group in cIMS^2^. Additional fragmentation in cIMS^3^ shows that the loss of AgH provides product ions at *m/z* 575.5034 that have identical drift times. Thus, in accordance with previous theoretical data [32], this suggests that the cIMS^3^ product ion at *m/z* 575.5034 has a similar shape for both isomers PC 16:0/18:1 and PC 18:1/16:0 after the loss of AgH. Despite the similarity of the MS^3^ product ions displayed in the driftogram (Fig. S4a and S4b), the two isomers are still different in MS^3^ since unique fragments are formed in MS^4^ for the respective isomer as discussed below. This suggests that the position of the residual charge induced by AgH loss is imperative for generating *sn-*positional isomer-specific product ions in MS^4^.

The structure of the MS^3^ product ion at *m/z* 575.5034 includes a dioxolane ring and a C = C double bond to the *sn*-2 fatty acyl chain (Fig. 1c and d) [40]. Despite the identical drift time and shape of the isomers PC 16:0/18:1 and PC 18:1/16:0, additional fragmentation in MS^4^ produces diagnostic product ions through cleavages at the five-membered 1,3-dioxolane ring. This dissociation specifically provides a base peak in MS^4^ that is unique for the *sn*-2 acyl chain. For the positional isomers PC 16:0/18:1 and PC 18:1/16:0, the base peaks are found at *m/z* 263.2369 and *m/z* 237.2213, respectively (Fig. 1d), which correspond to 18:1 and 16:0 *sn*-2 acyl chains. The *sn*-1 acyl chain is also found in the MS^4^ spectrum, but at levels down to ~ 5% of the *sn*-2 signal (Fig. 1d). The significantly higher dissociation of the acyl chain in *sn-*2 position suggests that the connectivity between the acyl chain in the sn-1 position is much more stable. The detected acyl chain product ions can there readily be used to annotate the *sn*-positional isomers.

The selected precursor limits the potential acyl chain combinations to a set of carbons and double bonds. For example, using the precursor PC 36:1, some theoretical acyl chain combinations are 16:0_20:1, 16:1_20:0, 18:0_18:1, and 18:1_18:0, which can be corroborated experimentally by detecting the corresponding acyl chain fragments in MS^4^. The presence and *sn*-position of potential acyl chain pairs of the selected precursor are determined using the formula C_x_H_2x-1-2n_O^+^ (*n* = number of double bonds) for the *sn*-1 chain and C_x_H_2x-3-2n_O^+^ for the *sn*-2 chain. For example, the product ions for PC 16:0/18:1 will be detected at *m/z* 239.2370 and *m/z* 263.2369 for *sn*-1 and *sn*-2, respectively, while keeping in mind that the *sn*-2 is more likely formed as further discussed in the quantitation section below. By determining the acyl chains in MS^4^, their distinct *sn*-positions are mapped and the *sn*-positional isomers of the precursor annotated. Interestingly, the method was also applied successfully to the fully saturated PC 13:0_12:0, showing that unsaturations in the acyl chains are not necessary for this method (Fig. S5). Overall, these results show that the silver adduct ions of PC enable rapid, specific, and unique *sn*-positional product ion formation in MS^4^ that can be readily interpreted.

### Annotation and quantitation of sn-positional isomers

**Fig. 2:**
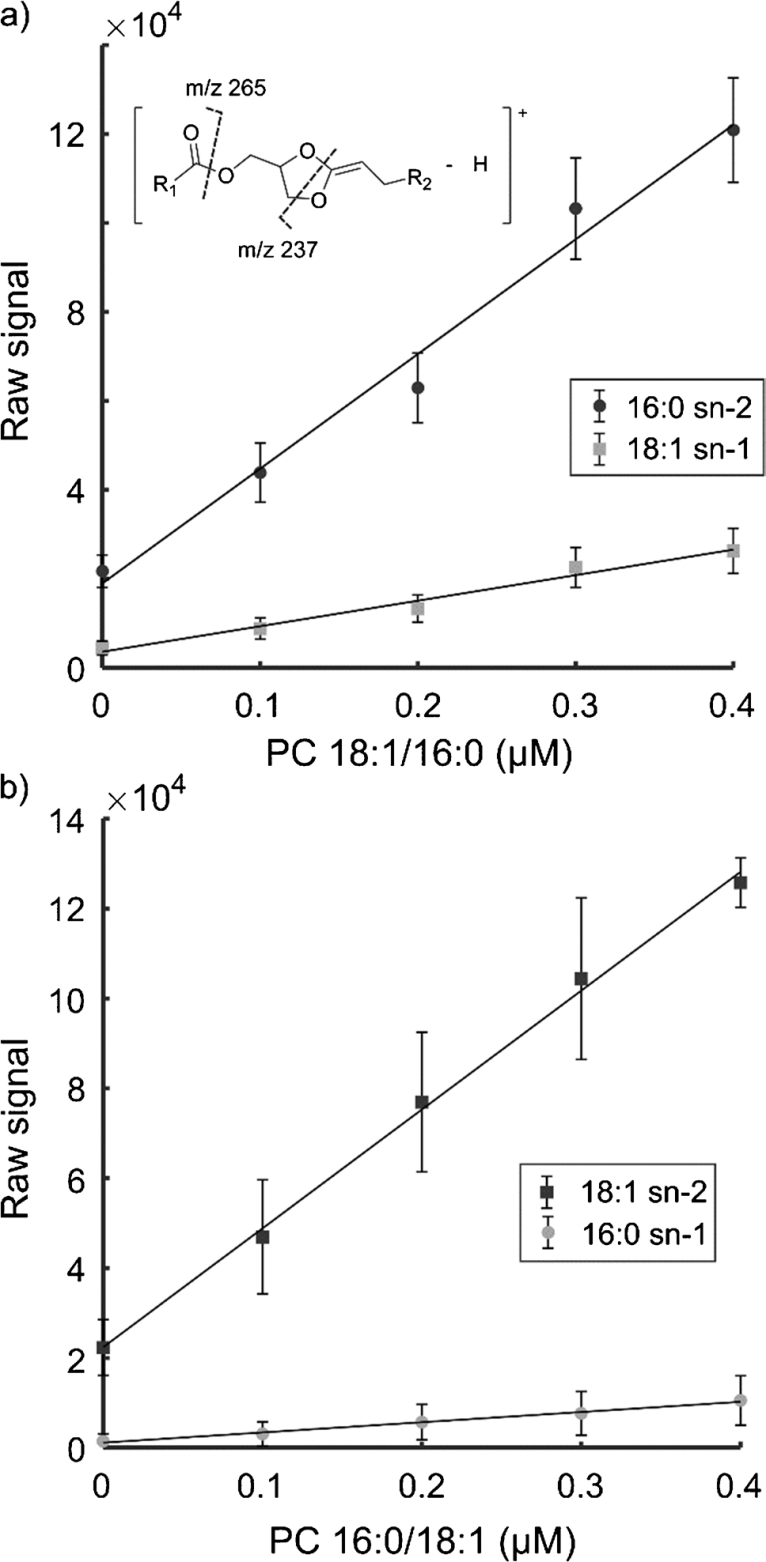
Calibration curves of mixtures of *sn*-positional isomer standards. **a** The two diagnostic product ions for the 16:0 in *sn*-2 position (black, *y* = 2.58E5*x* + 1.9E4, *r*^2^ = 0.983) and the 18:1 in *sn*-1 position (gray, *y* = 5.75E4*x* + 3.59E3, *r*^2^ = 0.978) acquired from standard solutions of 0.0–0.4 µM PC 18:1/16:0 and 1.0 µM PC 16:0/18:1. The insert shows the sites of fragmentation. **b** The two diagnostic product ions for the 18:1 in *sn*-2 position (black, *y* = 2.65E5*x* + 2.24E4, *r*^2^ = 0.997) and the 16:0 in *sn*-1 position (gray, *y* = 2.28E4*x* + 1.14E3, *r*^2^ = 0.992) acquired from standard solutions of 0.0–0.4 µM PC 16:0/18:1 and 1.0 µM PC 18:1/16:0. Error bars represent one standard deviation

The fragmentation efficiency into *sn*-1 and *sn*-2 product ions is vastly different. Specifically, the different abundances of the *sn*-1 and *sn*-2 diagnostic product ions in MS^4^ indicate that there are competing dissociation pathways that could also bias quantitation [13]. Calibration curves of the *sn*-specific product ions for PC 16:0/18:1 and PC 18:1/16:0 show a linear correlation for both *sn*-1 and *sn*-2 acyl chains (Fig. 2a–b). Additionally, the calibration curves show that the fragmentation efficiency for the *sn*-2 product ion is much higher for all concentrations, as indicated by the results in Fig. 1d. The slopes for the *sn*-2 product ions are close to identical, 2.58E5 for the 16:0 and 2.65E5 for the 18:1, but the slopes are substantially different for the *sn*-1 product ion, at 5.75E4 and 2.28E4 for the 18:1 and 16:0, respectively (Fig. 2). Importantly, the ratio between the *sn*-1 and *sn*-2 product ions is constant for each isomer (Fig. S6). Therefore, quantitation is possible when accounting for the relative fragmentation efficiency (*f*_*a*_) for the *sn*-1 and *sn*-2 product ions (Eq. 1), where the signal for the *sn*-2 acyl chain in MS^4^ (S_*sn*-2_) is divided by the sum of the signals from the *sn*-1 and *sn*-2 acyl chain pair in MS^4^ (S_*sn*-1_ + S_*sn*-2_).

1 $${\mathrm f}_{\mathrm a}={\mathrm S}_{\mathrm{sn}\text{-}2}/\left({\mathrm S}_{\mathrm{sn}\text{-}1}+{\mathrm S}_{\mathrm{sn}\text{-}2}\right)$$

Calculations show that the relative fragmentation efficiency for the *sn*-2 diagnostic product ion of PC 16:0/18:1 and PC 18:1/16:0 is 93% and 82%, respectively (Figs. S5 and S6). Interestingly, this suggests that when the *sn*-2 position contains an unsaturated acyl chain it is more likely to retain the charge, which could then be situated on the C = C bond, and form the *sn*-2 product ion. According to Kirschbaum et al. [40], the charge is located on the dioxolane ring for the precursor ion formed in MS^3^. Therefore, we hypothesize that the complexation between silver and the unsaturated acyl chain is a minor contributor to the MS^3^ product ion population. Therefore, we argue that the major fragmentation pathway is dependent on charge localization to the dioxolane ring. Despite the differences in fragmentation efficiency, the relative fragmentation efficiency can be used as a factor for the detected signal to quantify the isomeric fraction, as shown in Eq. 2.

2 $$\mathrm{Isomeric}\;\mathrm{fraction}=\frac{\frac{{\mathrm S}_{\mathrm a}}{{\mathrm f}_{\mathrm a}}}{\sum\frac{{\mathrm S}_{\mathrm x}}{{\mathrm f}_{\mathrm x}}}\ast100$$

Equation 2 states that the quantitative isomeric fraction equals the ratio of the *sn*-2 isomer signal (*S*_a_) and its fragmentation efficiency (*f*_*a*_, Eq. 1) divided by the sum of all detected *sn*-2 signals originating from the same precursor ion over their respective fragmentation efficiency (S_x_/f_x_). Thus, the isomeric fraction can be used to enable quantitation without bias.

**Fig. 3:**
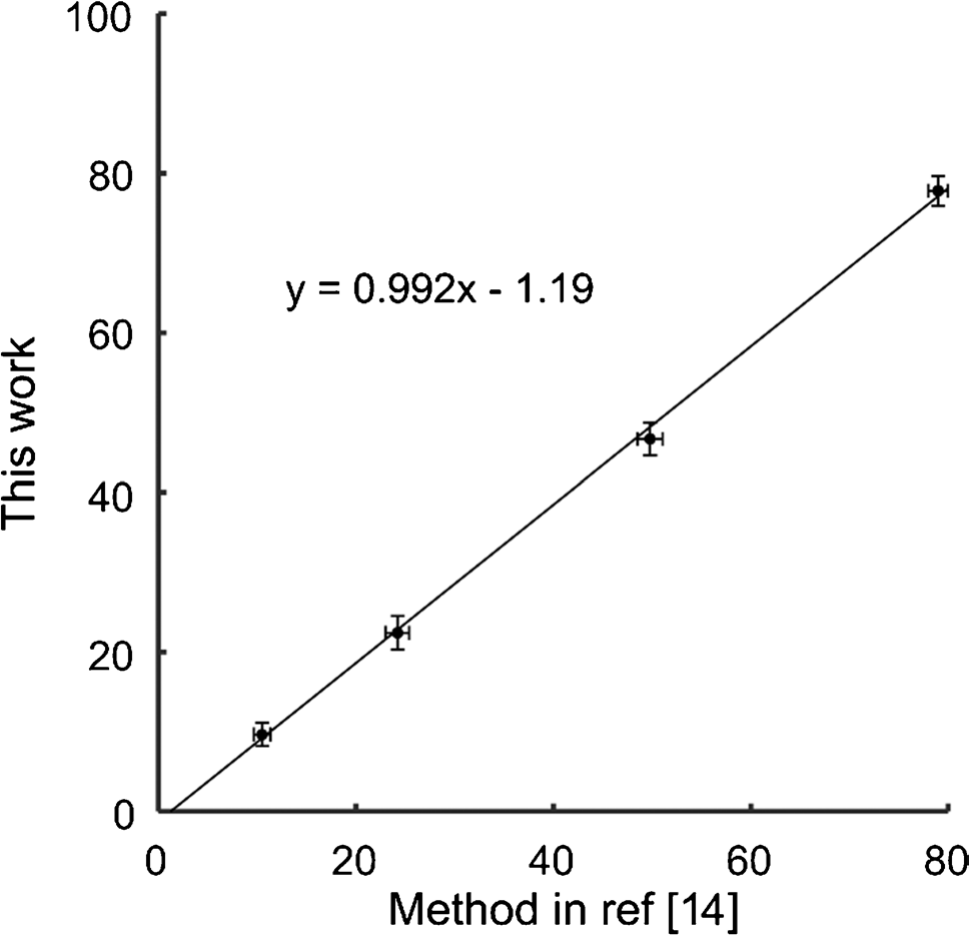
Comparison of the predicted isomeric fraction of PC 16:0/18:1 in standard mixtures by this work (*y*-axis) and the method presented in Ekroos et al. [14] (*x*-axis). The two methods yield very similar results; the regression line has a slope of 0.992 with an intercept at − 1.19. Error bars represent one standard deviation

The developed quantitative approach was validated against the previously reported method from Ekroos et al. [14], which is based on acetate adduct formation to generate unique product ions in MS^n^. For direct comparison, the two methods were run in parallel using the same original standard solutions of PC 16:0/18:1 and PC 18:1/16:0 and with all other parameters adapted to the respective optimal method conditions. Following, the quantitative results were compared and found to correlate almost perfectly (Fig. 3). The linear regression line is *y* = 0.992*x* − 1.19 and both approaches show similar and low (below 5%) coefficients of variations despite the large difference in methodology. In addition, we observed a similar sensitivity between the two methods. Both our method and the method by Ekroos et al. [14] enabled the determination of *sn*-positional isomer concentrations in the nM–µM range, which is up to 100 times better than previous reports using UVPD, PB, and OzID, although it can potentially be pushed lower for all methods [19, 20, 41]. Since similar structures are formed in these methods, the results indicate that silver is necessary to form the dioxolane carbocation structure and enable charge retention for dissociation in MS^4^ by reduction of the silver ion in previous dissociation steps. In addition, our preliminary results show that the same structures are formed for all glycerolipids. Overall, silver cationization provides both a sensitive and a quantitative method for the determination of *sn*-positional isomers in positive ion mode.

### Mass spectrometry imaging of sn-positional isomers in mouse brain

The sensitivity afforded by silver cationization indicates its applicability for quantitative analysis of chemically complex biological samples and mass spectrometry imaging. Mass spectrometry imaging provides insights into the distribution of molecules in thin tissue sections and aids in understanding chemical processes and actions in morphological regions. Previous publications determining the distribution of *sn*-positional isomers of PC with MSI have shown that the relative abundance of *sn*-positional isomers varies between tissue regions [16, 17, 23]. Specifically, the molar fraction of PC isomers with unsaturated acyl chains in the *sn*-2 position has been reported higher in the white matter compared to the gray matter regions of the mouse brain [16, 17, 23]. However, to compare amounts of individual PC isomers for insights into biological processes, a quantitative approach is necessary. Quantitative differential distributions of *sn*-positional isomeric species of PC 34:1 and PC 36:1 in mouse brain were mapped by doping the solvent with silver and PC 25:0 for subsequent imaging using nano-DESI in MS^n^ [45–47]. The method was incorporated into a regular MSI workflow by interlacing the MS^4^ scan events in the ion trap with the FTMS MS^1^ full scan event for online quantitation. The MS^1^ and MS^4^ scan events were spaced by 5 µm for PC 34:1 and 15 µm for PC 36:1 in the tissue, thus adding about 1 s to the duty cycle. Pixel sizes of 60 by 75 µm were achieved by oversampling, which is comparable with reported pixel sizes from other MSI studies [16, 17, 23, 35]. Quantitation was performed according to Eq. 3, where the detected concentration of the isomer *a* (*c*_*a*_) is calculated using its precursor signal (*S*_*A*_) in the MS^1^ scan event divided by the detected signal for the internal standard (*S*_*IS*_) in the same MS^1^ scan. Furthermore, it is multiplied by the known concentration of the internal standard (*c*_*IS*_), the type 1 carbon correction factor (*Z*) (described in Eq. S1), and the *Isomeric fraction* provided by Eq. 1.

3 $${\mathrm c}_{\mathrm a}=\frac{{\mathrm S}_{\mathrm{A\ MS}^1}}{{\mathrm S}_{\mathrm{IS\ MS}^1}}{\mathrm c}_{\mathrm{IS}}\ast\mathrm Z\ast\mathrm{Isomeric}\;\mathrm{fraction}$$

**Fig. 4:**
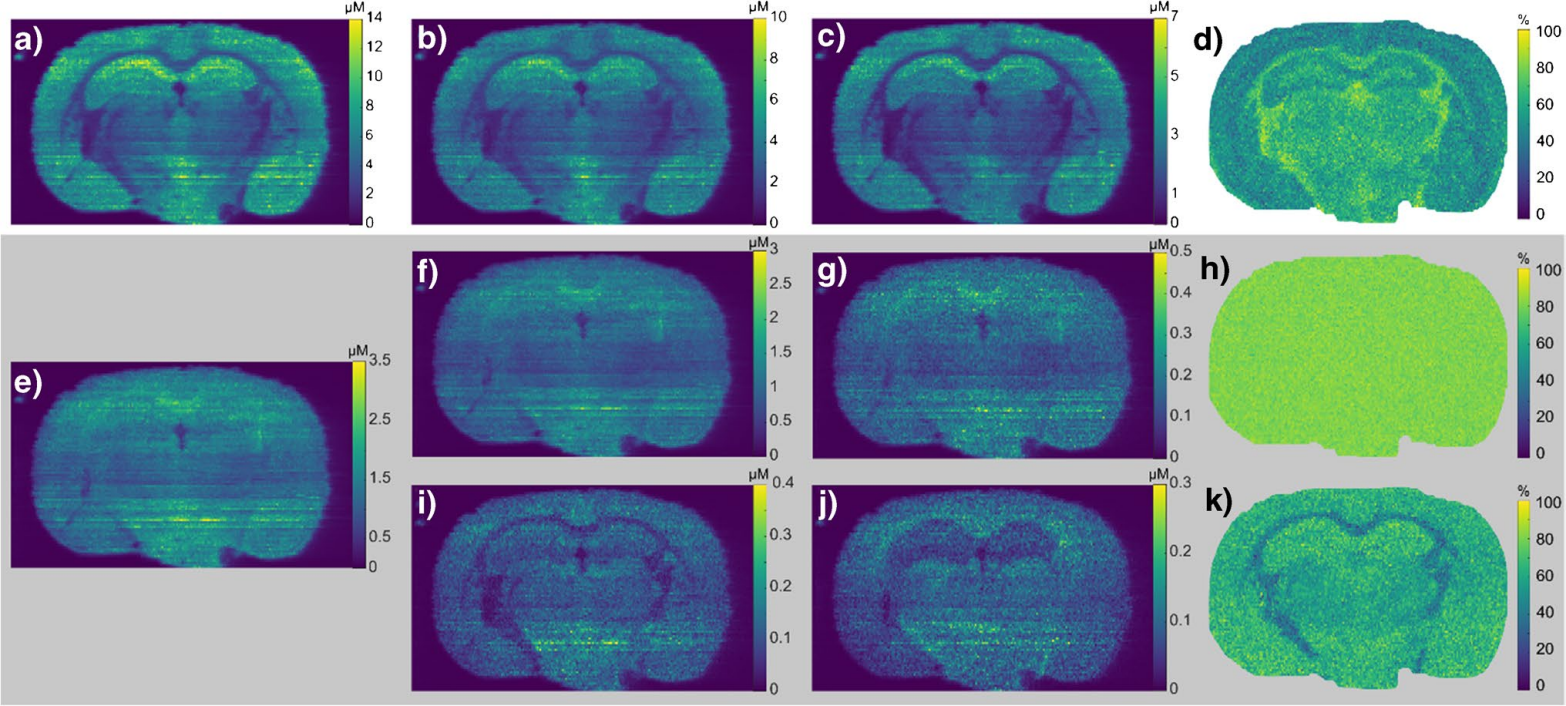
Ion images of a mouse brain tissue section showing the precursors and their *sn*-positional isomers generated using silver-doped quantitative nano-DESI MSI ion images. **a** Quantitative ion image of PC 34:1 from the MS^1^ scan event. **b** Quantitative ion image of PC 16:0/18:1. **c** Quantitative ion image of PC 18:1/16:0. **d** Isomeric fraction of PC 16:0/18:1 to PC 18:1/16:0. **e** Quantitative ion image of PC 36:1 from MS^1^. **f** Quantitative ion image of PC 18:0/18:1. **g** Quantitative ion image of PC 18:1/18:0. **h** Isomeric fraction of PC 18:0/18:1 to PC 18:1/18:0. **i** Quantitative ion image of PC 20:1/16:0. **j** Quantitative ion image of PC 16:0/20:1. **k** Isomeric fraction of PC 20:1/16:0 to PC 16:0/20:1. All ion images of the isomers are generated in MS^4^

For the targeted PC 34:1, two *sn*-positional isomers were detected, specifically PC 16:0/18:1 and PC 18:1/16:0. Despite having large differences in overall concentrations, as revealed by the quantitative scaling, they have a nearly identical spatial distribution that mirrors the precursor PC 34:1 (Fig. 4a–c). Note that the ability to generate quantitative ion images of *sn*-positional isomers is unique for this study. The more commonly used isomeric fraction ion image generated as a ratio of PC 16:0/18:1 and PC 18:1/16:0 [23] highlights that there are slight differences between their locations that are not visible when comparing the individual ion images (Fig. 4d). In particular, the ratio image shows that the distribution is similar to previously published works, which report 31 ± 2 and 43 ± 2% in the white and gray matter respectively of PC 16:0/18:1 [23], whereas our results show 39 ± 3% and 46 ± 3% in the same regions when using the quantitative approach. These results are similar and the slight deviation is attributed to the difference between the intensity ratio and the quantitative ratio.

The precursor PC 36:1 was found to include two *sn*-positional isomer pairs, namely PC 18:1/18:0 and PC 18:0/18:1, and PC 16:0/20:1 and PC 20:1/16:0. The most abundant isomer pair is the PC 18:0/18:1 and PC 18:1/18:0, and these are therefore the main contributors to the distribution of the precursor PC 36:1 ion image (Fig. 4e–g). Note that the fragmentation efficiency of PC 18:1/16:0 was used for the PC 18:1/18:0 since these product ions have the same *m/z* value (Fig. S7). Interestingly, the two isomers PC 18:0/18:1 and PC 18:1/18:0 are equally distributed in all regions of the mouse brain as revealed by their isomeric fraction ion image (Fig. 4h). Contrarily, the isomer pair PC 20:1/16:0 and PC 16:0/20:1 does not mirror the distribution of the precursor and show individual and inverted distributions in the CC and the hippocampus (Fig. 4i and j). These specific distributions are hidden under the total signal from the precursor unless the isomers are targeted. The individual distributions of the isomer pair become increasingly clear in the isomer ratio ion image (Fig. 4k), in agreement with the previous report [23]. The ability to generate quantitative ion images of each *sn*-positional isomer in addition to the ratio image and the precursor ion image is unique for this study and we anticipate that the exact isomer locations and abundance will provide additional dimensions when studying biological systems.

**Fig. 5:**
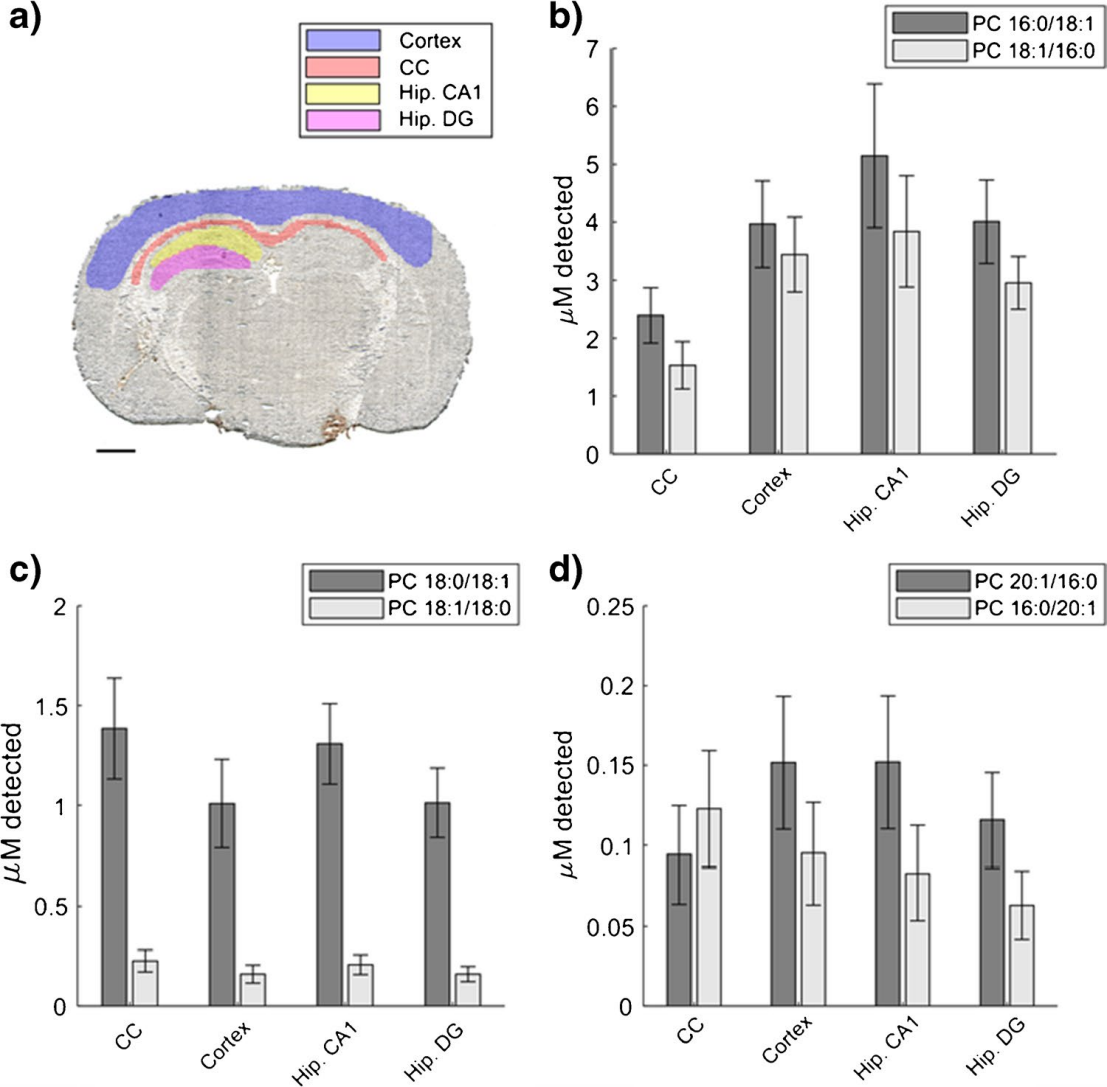
Region of interest analysis of four selected regions in the mouse brain tissue displayed in **a** optical image with 1 mm scale bar, with highlighted regions and number of pixels (*n*) in each region, cortex (blue, *n* = 1844), corpus callosum (CC) (red, *n* = 434), sub-hippocampal region CA1 (yellow, *n* = 331), and sub-hippocampal region dentate gyrus (DG) (pink, *n* = 355). **b**–**d** Detected concentrations of **b** PC 16:0_18:1 isomers, **c** PC 18:0_18:1 isomers, **d** PC 20:1_16:0 isomers. The error bars represent one standard deviation of the averaged concentration in the region of interest

The quantitative strategy enables in-depth characterization of the abundance of selected isomers in morphological regions using region of interest (ROIs) analysis. The regions selected from the mouse brain tissue include the cortex, corpus callosum (CC), and the two sub-hippocampal regions CA1, and dental gyrus (DG) (Fig. 5a). A comparison of the CC and cortex regions for the isomer pair PC 16:0/18:1 and PC 18:1/16:0 originating from PC 34:1 shows that the detected concentration of PC 16:0/18:1 is 0.86 µM and 0.52 µM higher than PC 18:1/16:0 (Fig. 5b). Furthermore, the highest concentrations of both isomers are found in the CA1 while the concentration in DG is similar to the cortex. The precursor PC 36:1 has two isomer pairs, and the ROI data reveal that despite the identical distribution of PC 18:0/18:1 and PC 18:1/18:0 (Fig. 4h), the PC 18:0/18:1 is the dominating isomer with almost 1.5 µM detected in the CC and CA1 regions (Fig. 5c). On the contrary, the isomer pair PC 20:1/16:0 and PC 16:0/20:1 is detected in low sub-µM concentration and minute regional difference in concentration (Fig. 5d). Specifically, the concentration is 0.028 µM higher for PC 16:0/20:1 in the CC, whereas in the cortex, CA1, and DG of the hippocampus, there is 0.057, 0.069, and 0.053 µM more PC 20:1/16:0, respectively. Previous studies have suggested that the spatial distribution of *sn*-positional isomers is driven by distinct activities of PLA enzymes in different regions [23]. However, because there is no differential distribution of PC 18:1_18:0 detected, we argue that the activities of PLA enzymes are not driving the specific localization. Instead, we suggest that the specific properties of these individual isomers are necessary for cellular and protein function in these regions and therefore their abundances are precisely regulated.

## Conclusions

In this study, we report a new method for annotation and quantitation of *sn*-positional isomers of glycerophospholipids using silver cationization. Silver cationization in combination with MS^n^ and loss of AgH provides diagnostic product ions of *sn*-positional isomers. The developed method is readily adaptable to any mass spectrometric system that has the ability to perform MS^n^ by simply adding silver to the sample solution. The obtained sensitivity is in the nM–µM range, which is comparable with the previously highest reported sensitivity. Furthermore, the annotation and distribution of multiple *sn*-positional isomers from PC 34:1 and PC 36:1 mirror previous findings, with the addition of providing the detected concentration for each isomer. We envision that the method can be directly applicable to all glycerolipids and that its simplicity, sensitivity, and ability for quantitation will provide a new benchmark in the analysis of *sn*-positional isomers.

## Supplementary Information

Below is the link to the electronic supplementary material.

Supplementary file1 (DOCX 919 KB)
