# Supplementary material for: Quantitative determination of sn-positional phospholipid isomers in MSn using silver cationization
Source: Anal Bioanal Chem. 2022 Jun 22;414(25):7473–82. doi: 10.1007/s00216-022-04173-6 (PMC9482905; doi:10.1007/s00216-022-04173-6)
Supplement: Supplementary file 1 — Supplementary file1 (DOCX 919 KB) [file 216_2022_4173_MOESM1_ESM.docx]

**Supporting information**

Quantitative determination of *sn*-positional phospholipid isomers in MS^n^ using silver cationization

Johan Lillja, Ingela Lanekoff

Department of Chemistry – BMC, Uppsala University, Uppsala, Sweden

Corresponding author:

Prof. Ingela Lanekoff

Ingela.Lanekoff@kemi.uu.se

Dept. of Chemistry-BMC (576)

Uppsala University

751 23 Uppsala

Sweden

Table of Contents

[Figure S1 MS^n^ transitions for PC 16:0/18:1 3](#_Toc103154682)

[Figure S2 MS^3^ product ion spectra of [FA+Ag]^+^ type ions of PC 16:0/18:1 and PC 18:1/16:0 3](#_Toc103154683)

[Figure S3 MS^3^ product ion spectra for PC 34:1 ionized with ^109^Ag and ^107^Ag repsectively 4](#_Toc103154684)

[Figure S4 Driftograms for the cIMS^n^ data 4](#_Toc103154685)

[Figure S5 MS^n^ Transitions for PC 13:0_12:0 5](#_Toc103154686)

[Figure S6 Fragmentation efficiency of PC 18:1/16:0 and PC 16:0/18:1 6](#_Toc103154687)

[Figure S7 Fragmentation efficiency for PC 18:1_16:0 and PC 20:1_16:0 measured from tissue 7](#_Toc103154688)

[Scheme S1 Fragmentation scheme 8](#_Toc103154689)

[Equation S1 Type 1 correction factor 8](#_Toc103154690)


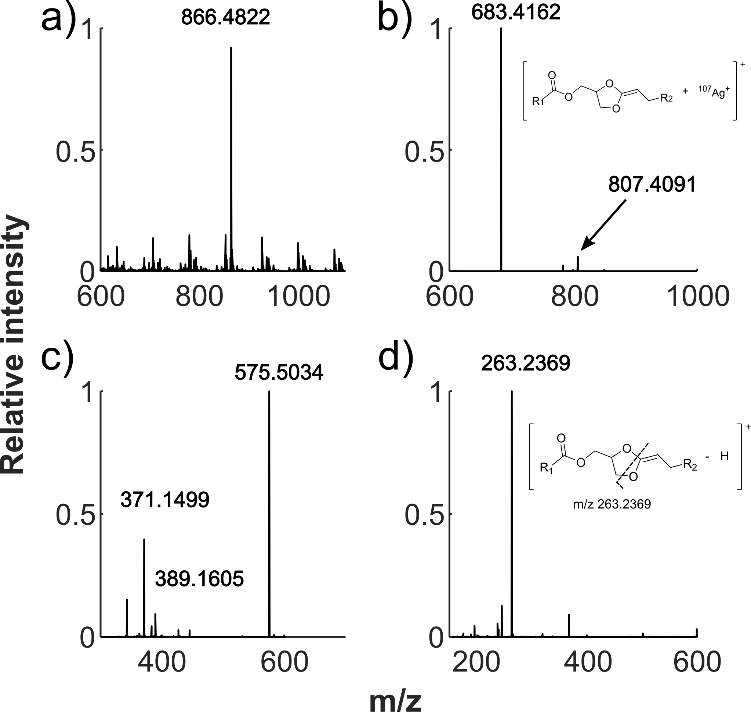


Figure S1 MS^n^ transitions for PC 16:0/18:1 Tandem mass spectrometric transitions of PC 16:0/18:1 a) MS^1^ spectrum showing the [M+H]^+^ ion at *m/z* 760.5839 and [M+Ag]^+^ at *m/z* 866.4822 b) MS^2^ spectrum of the [M+Ag]^+^ peak giving product ions corresponding to choline loss at *m/z* 807.4091 and head group loss at *m/z* 683.4162 c) MS^3^ spectrum using 683.4162 as precursor ion, where the main product ion at *m/z* 575.5034 corresponds to a NL of AgH and the other annotated ions represent acyl chain losses and d) MS^4^ of the 575.5034 ion giving diagnostic product ions with respect to the *sn* position.


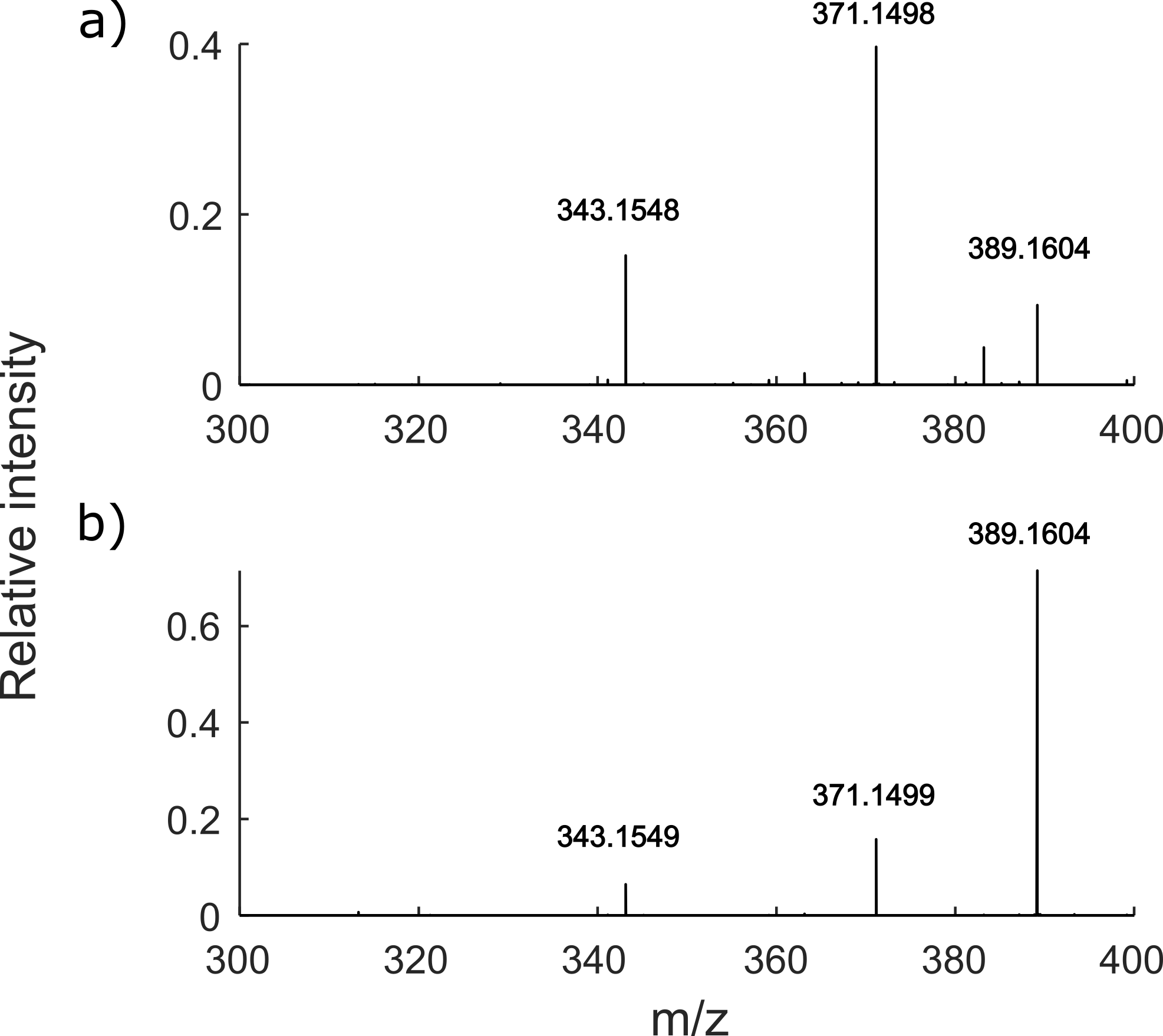


Figure S2 MS^3^ product ion spectra of [FA+Ag]^+^ type ions of PC 16:0/18:1 and PC 18:1/16:0 Product ions in MS^3^ from isomers of a) PC 16:0/18:1 and b) PC 18:1/16:0 with *m/z* 389.1604 ([FA 18:1 + ^107^Ag]^+^) , 371.1498 ([FA 18:1 + ^107^Ag - H_2_O]^+^) and 343.1549 ([FA 18:1 + ^107^Ag - H_2_O - CO]^+^) formed from both PC 18:1/16:0 and PC 16:0/18:1 isomers. The ions are not unique for the respective isomer and can therefore not be used directly for quantification.


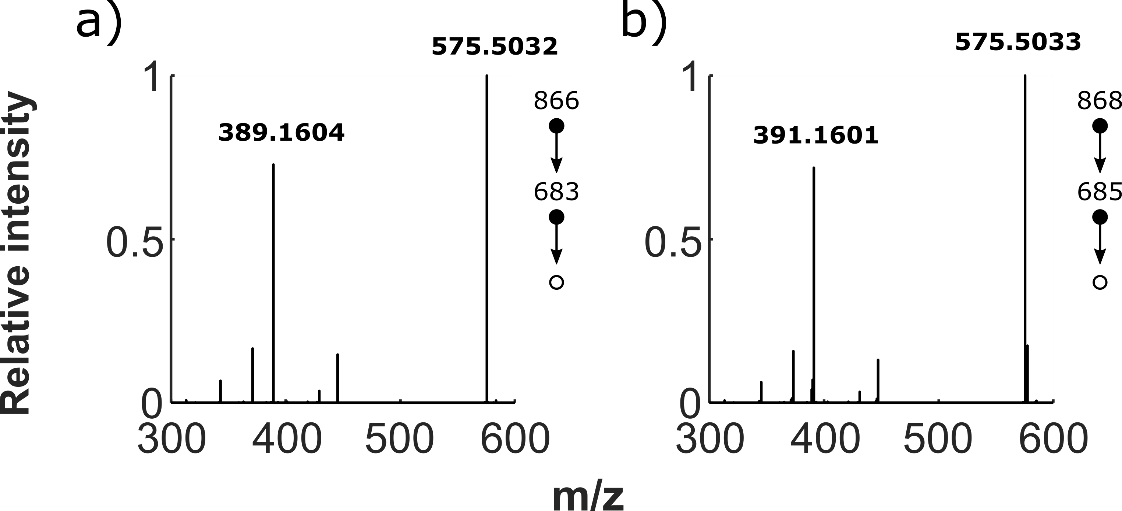


Figure S3 MS^3^ product ion spectra for PC 34:1 ionized with ^109^Ag and ^107^Ag repsectively a) MS^3^ spectrum of [M+^107^Ag]^+^ showing the –AgH peak at *m/z* 575.5032 and [FA+^107^Ag]^+^ at *m/z* 389.1604 b) MS^3^ spectrum of [M+^109^Ag]^+^ showing the –AgH peak at *m/z* 575.5033 and [FA+^109^Ag]^+^ at *m/z* 391.1601. The difference in mass between ^107^Ag^+^ and ^109^Ag^+^ is 1.9997 Da, which equals the difference in mass of [FA+^107^Ag]^+^ at *m/z* 389.1604 and and [FA+^109^Ag]^+^ at *m/z* 391.1601.


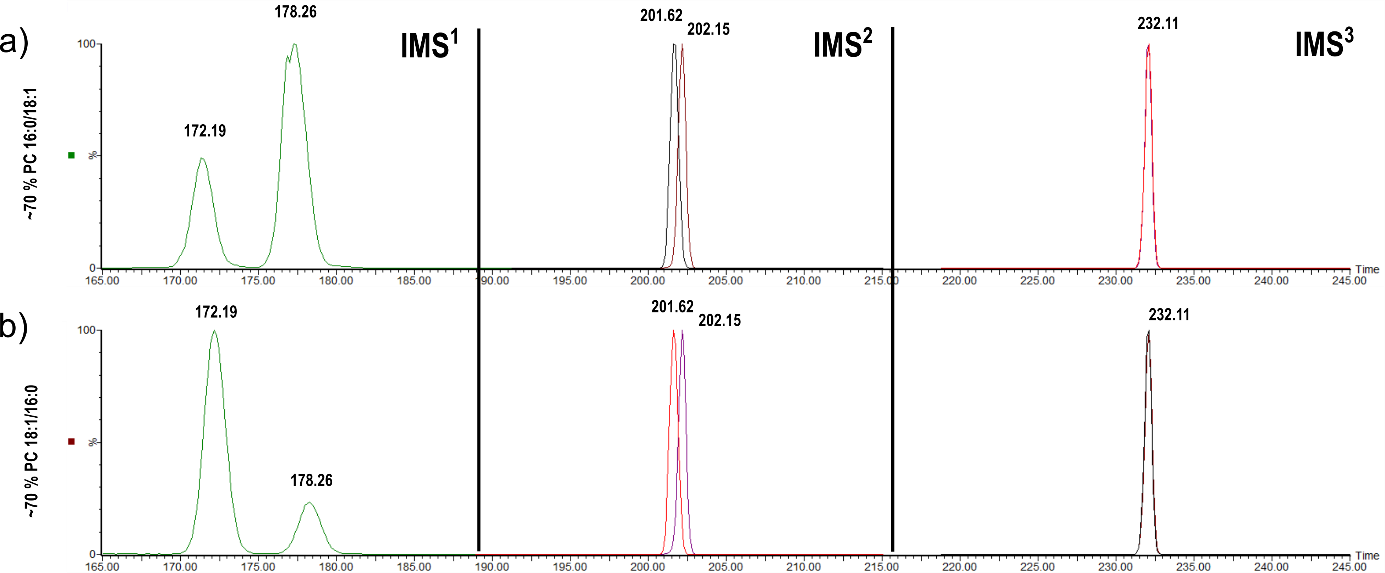


Figure S4 Driftograms for the cIMS^n^ data Driftogram of the ions in this fragmentation pathway in IMS^1^, IMS^2^ and IMS^3^. A) Direct infusion of 1 µM PC 16:0/18:1 spiked with 0.4 µM PC 18:1/16:0 in IMS^1^ with 10 passes in the cyclic IMS (cIMS). The drift time of the *sn*-1 isomer is 172.19 ms and 178.26 ms for the *sn*-2 isomer. In IMS^2^ the separated isomers from IMS^1^ were used as precursors. In the driftogram of *m/z* 683.4204 the *sn*-1 isomer product ion has a drift time of 201.62 ms wheras the product ion of the *sn*-2 isomer has a drift time of 202.15 ms using one pass in the cIMS device. In IMS^3^ the precursor was chosen in the same way and the driftogram of the product ion at *m/z* 575.5067 is shown with the drift time 232.11 ms using one pass in the cIMS device for both isomers. B) 1 µM PC 18:1/16:0 spiked with 0.4 µM PC 18:1/16:0 in IMS^1^ the drift time of the *sn*-1 isomer is 172.19 ms and 178.26 for the *sn*-2 isomer with 10 passes in the cIMS device. In IMS^2^ the separated isomers from IMS^1^ was used as precursors the driftogram of *m/z* 683.4204 where the *sn*-1 isomer product ion has a drift time of 201.62 ms wheras the product ion of the *sn*-2 isomer has a drift time of 202.15 ms with one pass in the cIMS device. In IMS^3^ the precursor was chosen in the same way and the driftogram of the product ion at *m/z* 575.5067 is shown with the drift time 232.11 with one pass in the cIMS device for both isomers.


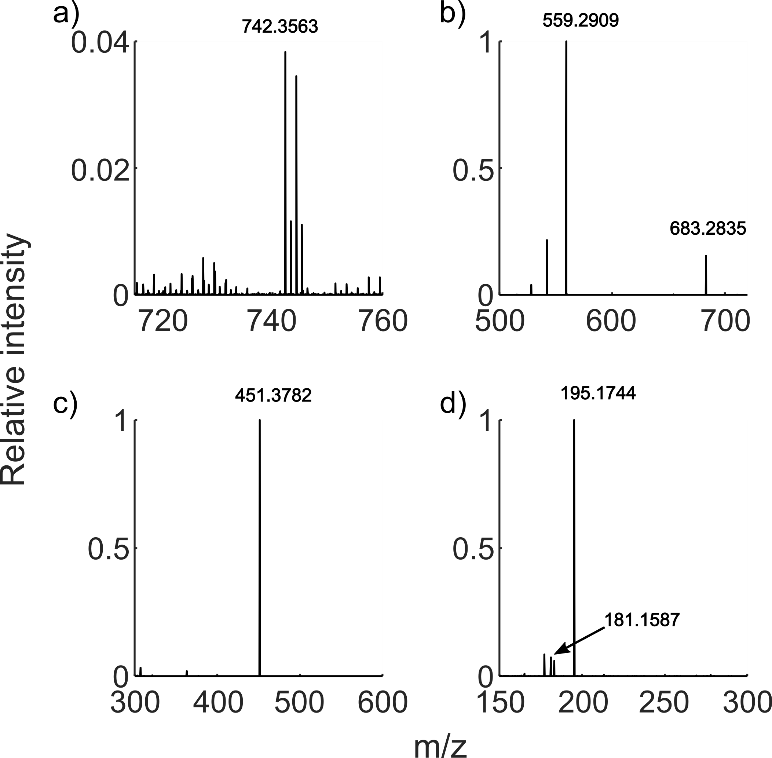


Figure S5 MS^n^ Transitions for PC 13:0_12:0 Tandem mass spectrometric transitions of PC 13:0_12:0 a) MS^1^ spectrum showing the [M+Ag]^+^ at *m/z* 742.3563 b) MS^2^ spectrum of the [M+Ag]^+^ peak giving product ions corresponding to choline loss at *m/z* 683.2835 and head group loss at *m/z* 559.2909 c) MS^3^ spectrum using 559.2909 as precursor ion, where the main product ion at *m/z* 451.3782 corresponds to a NL of AgH d) MS^4^ of the 451.3782 ion giving diagnostic product ions with respect to the *sn* position of the 12:0 and 13:0 acyl chains.


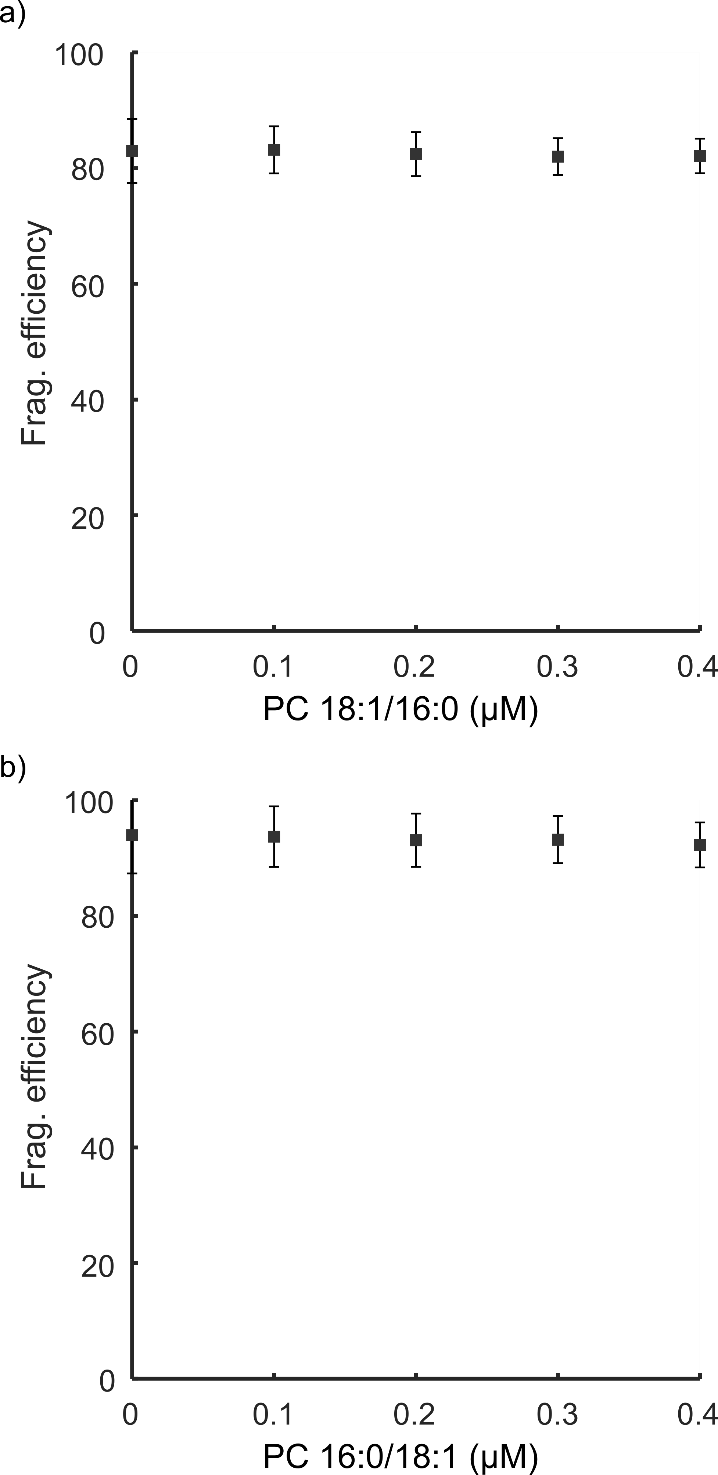


Figure S6 Fragmentation efficiency of PC 18:1/16:0 and PC 16:0/18:1 Fragmentation efficiency for the *sn*-2 type product ion from 0 - 0.4 µM a) fragmentation efficiency for the 16:0 in *sn*-2 product ion (*m/z* 237) was estimated to 82% ± 3.5 % b) fragmentation efficiency for the 18:1 in *sn*-2 product ion (*m/z* 263) was estimated to 93 % ± 4 %. Error bars represent one standard deviation.


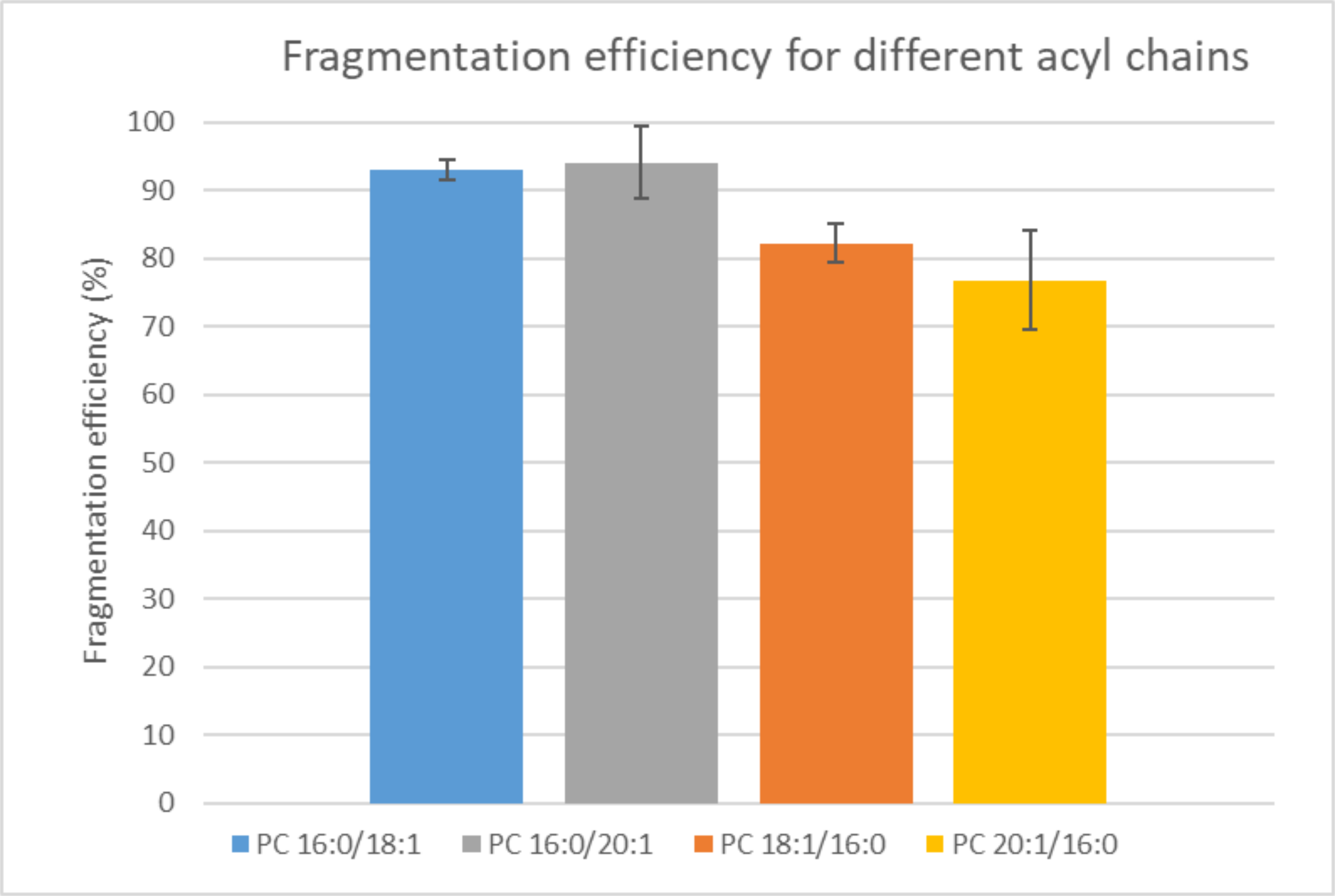


Figure S7 Fragmentation efficiency for PC 18:1_16:0 and PC 20:1_16:0 measured from tissue The fragmentation efficiency of PC 16:0/18:1 was 93 ± 1.5 %, PC 16:0/20:1 was 94 ± 5 %, PC 18:1/16:0 was 82 ± 3 %, and PC 20:1/16:0 was 77 ± 7 %. The observed fragmentation efficiency mirrors the measured efficiency in standards, which was 93 ± 4 % and 82 ± 3.5 for PC 16:0/18:1 and PC 18:1/16:0 respectively. Error bars represent one standard deviation.


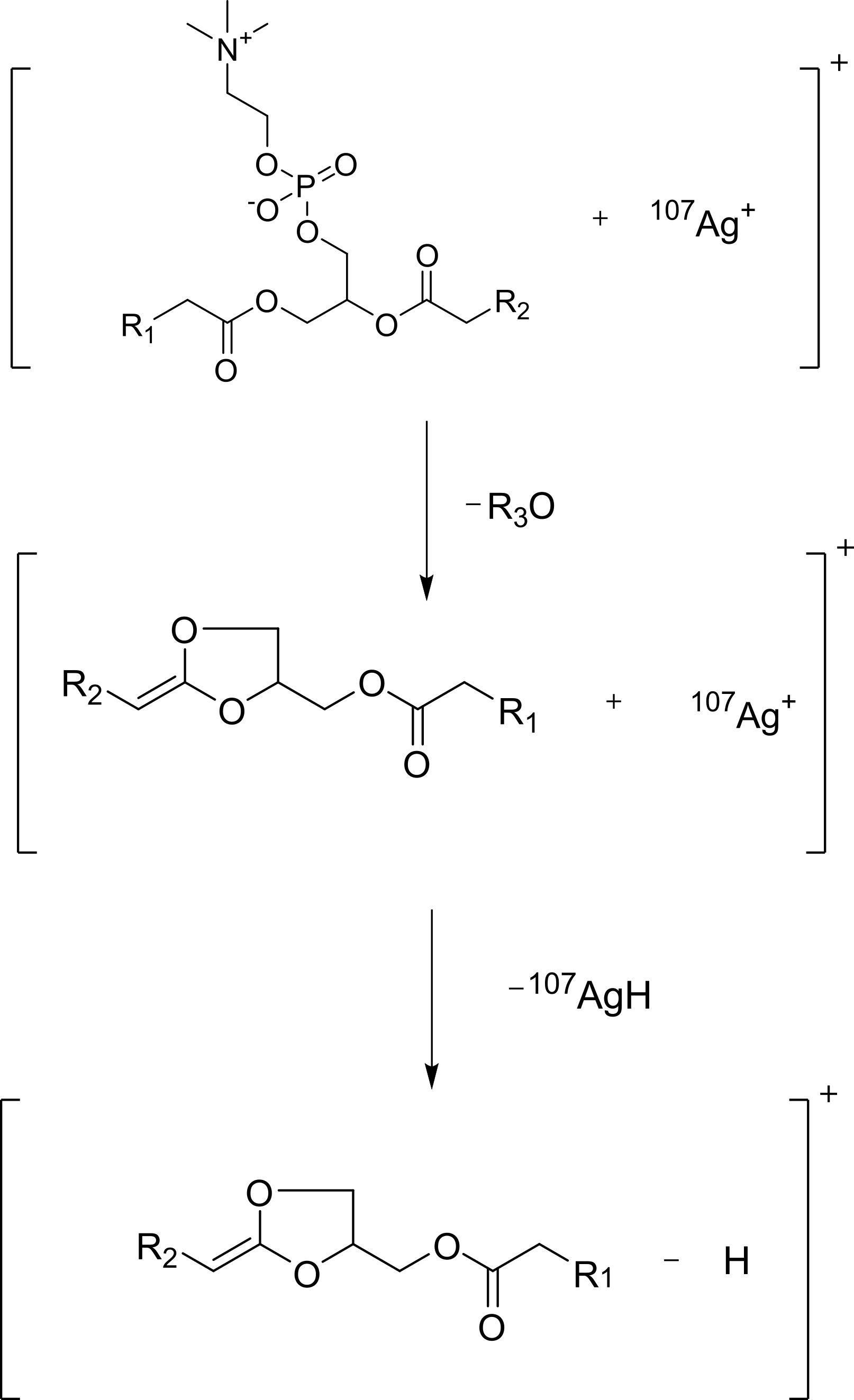


Scheme S1 Fragmentation scheme General fragmentation scheme of a glycerolipid where R_1_ and R_2_ are acyl chains and R_3_ is either an acyl chain or a modified phosphate *headgroup*.

$$Z=\frac{1+0.0109n+\frac{{0.0109}^{2}n\left( n-1 \right)}{2}}{1+0.0109s+\frac{{0.0109}^{2}s\left( s-1 \right)}{2}}$$

Equation S1 Type 1 correction factor n is the number of carbons in the analyte and s is the number of carbons in the internal standard. 0.0109 is the probability of having a carbon 13 isotope in the analyte. [1]

1. Yang K, Han X (2011) Accurate quantification of lipid species by electrospray ionization mass spectrometry - Meets a key challenge in lipidomics. Metabolites 1:21–40. https://doi.org/10.3390/metabo1010021
